# How Does Place of Residence Influence the Experience and Management of Chronic Non‐Malignant Pain? A Systematic Review

**DOI:** 10.1002/ejp.70443

**Published:** 2026-11-02

**Authors:** Christiana Stylianou, Rosalind Adam, Fortune Kwushue, Barbara I. Nicholl

**Affiliations:** ^1^ School of Health & Wellbeing University of Glasgow Glasgow UK; ^2^ Academic Primary Care, Institute of Applied Health Sciences University of Aberdeen Aberdeen UK

## Abstract

**Background and Objective:**

Chronic non‐malignant pain (CNMP) affects up to half of the UK population and one third of adults globally. Rural populations experience poorer health outcomes across several chronic conditions; however, the influence of place of residence on CNMP management, outcomes and experiences remains unclear. This systematic review examined how place of residence (urban or rural) impacts CNMP management, outcomes and experiences.

**Methods:**

PubMed, psycINFO, Medline, Embase and CINAHL databases were searched for qualitative and quantitative studies. Dual title/abstract screening and full‐text review were undertaken against pre‐defined inclusion/exclusion criteria. A narrative synthesis was performed.

**Results:**

Of 2341 titles screened, 16 studies were included (12 observational, three qualitative and one mixed‐methods study). Most studies were rated as fair quality, with limitations including unsatisfactory response rates and non‐validated outcome measures. Rural residents were more likely to use primary care and opioid‐based treatments, whereas urban residents had greater access to specialist pain services. Outcomes were less consistent; several studies reported poorer pain‐related outcomes among rural residents, although some identified no significant differences compared with urban populations. Long travel distances, prolonged waiting times and limited specialist and non‐pharmacological services were barriers to care.

**Conclusions:**

This review highlights geographic inequalities in CNMP management and experiences. Findings should be interpreted in light of methodological limitations and study heterogeneity. Targeted approaches are needed to improve equitable access to pain management across settings.

**Significance Statement:**

This systematic review synthesises evidence on how geographic location influences the management, outcomes and experiences of people with chronic non‐malignant pain. The findings identify consistent rural–urban disparities in access to evidence‐based pain care and highlight important gaps in the current literature, including the limited availability of high‐quality comparative studies. These findings provide a foundation for future research and inform the development of more equitable pain services.

**Trial Registration:**

PROSPERO number: CRD42024522734

## 1 Introduction

Chronic pain is defined by the International Association of the Study of Pain (IASP) as “pain that persists or recurs for longer than three months” (IASP 2025). Chronic non‐malignant pain (CNMP) affects up to half of the population in the United Kingdom (UK) and approximately one third of adults globally (Fayaz et al. 2016). It represents a major public health and economic burden, with recent national guidance promoting evidence‐based multidisciplinary approaches to improve assessment, management, and quality of life (NICE 2021).

Chronic pain impacts significantly on wellbeing, quality of life, and functional capacity. It is the primary contributor to disability globally (Cohen et al. 2021) and is associated with sleep disturbance, depression and increased healthcare utilisation (Brown et al. 2011). People living with chronic pain are at substantially increased risk of suicidal ideation, suicide attempts and death by suicide compared with those without chronic pain (Kwon and Lee 2023). CNMP management places considerable demands on healthcare systems through medication prescribing, primary and secondary care consultations, diagnostic procedures and treatments, including surgery (NHSBSA 2025).

Health inequalities are well documented in the context of chronic pain with higher prevalence among individuals living in socioeconomic deprivation. There is also growing evidence of disparities across ethnic groups and between genders (Meints et al. 2019). However, rural–urban differences in the experience and management of CNMP are less well understood. Rural populations experience poorer health outcomes across several chronic conditions, including cancer (Carriere et al. 2018) and diabetes (Trivedi et al. 2011), with inequalities linked to healthcare workforce shortages, greater travel distances, reduced access to specialist services and wider barriers to healthcare access (Baker et al. 2024). To date, little is known about how place of residence (urban or rural) impacts on the management, outcomes and experience of CNMP.

Individuals living with CNMP in rural areas may face particular disadvantages. Chronic pain prevalence is higher in rural populations than in urban populations (Rafferty et al. 2021), potentially reflecting demographic differences, socioeconomic factors and physically demanding agricultural work. Furthermore, specialist pain services are predominantly located within secondary and tertiary care centres in urban regions (Suntai et al. 2021), while staffing shortages and regional variation further restrict access (Keyes et al. 2014; McGhie and Grady 2016). Telehealth has improved access for many patients, particularly since COVID‐19, but poor digital connectivity, limited opportunities for physical assessment, and challenges delivering multidisciplinary interventions may restrict its effectiveness in rural settings (Main et al. 2025).

Effective pain management requires a holistic biopsychosocial approach incorporating pain education and supported self‐management. As chronic pain can often be managed within community settings, rural residence should not inherently result in poorer outcomes. Nevertheless, emerging evidence suggests that rural populations may be prescribed opioids more frequently than urban populations (Keyes et al. 2014), raising questions about differences in management, healthcare access, patient experiences, and clinical outcomes.

This systematic review aims to investigate how place of residence (urban or rural) impacts the management, outcomes and experiences of people with CNMP.

## 2 Methods

A systematic review was conducted, following the Preferred Reporting Items for Systematic Reviews and Meta‐Analyses (PRISMA) guidelines (Page et al. 2021).

The study protocol was registered with PROSPERO (International Prospective Register of Systematic Reviews) under the registration number: CRD42024522734 (Stylianou et al. 2024).

The review sought to include both quantitative and qualitative literature to explore geographic disparities in the provisions and outcomes of CNMP management between urban and rural populations.

### 2.1 Search Strategy

After initial scoping of published literature, PubMed, PsycINFO, Medline, Embase and CINAHL databases were searched, using keywords and synonyms relating to chronic pain, pain management, rural health and urban health. The search strategy for Medline is presented in Appendix S1 of supplementary material. The electronic database searches for this systematic review covered the period from 1946 to 21st February 2024. An updated search covered the period from 22nd February 2024 to 2nd April 2025.

The results were supplemented by a manual search of the reference list of the included studies. In addition, studies from the authors' personal library identified from initial scoping prior to the literature search were included for screening.

### 2.2 Study Inclusion and Exclusion Criteria

Inclusion and Exclusion criteria (shown in Table 1) were devised following the Population Intervention Comparison Outcome (“PICO”) framework for systematic reviews. For the purpose of this review, CNMP was defined as pain persisting for 3 months or more that is not attributable to malignant disease.

**TABLE 1:** Inclusion/exclusion criteria.

Inclusion criteria	Exclusion criteria
Adults aged ≥ 18 years with chronic non‐malignant pain	Children or adolescents (< 18 years)
Study investigates the influence of place of residence (urban or rural) on pain management, outcomes or experience	Papers that do not investigate the influence of place of residence (urban or rural) on pain management, outcomes or experience
Study design: original research articles, qualitative studies, quantitative and observational studies (cross‐sectional/longitudinal)	Study design: systematic reviews, editorials, letters, conference proceedings and intervention studies
All clinical settings where chronic non‐malignant pain is managed	Papers evaluating individual interventions
Published in English language	Papers published in a language other than English

This review included studies that were published in English in any setting and country and (1) focused on adults aged 18 years or older with CNMP, (2) investigated the influence of place of residence (urban or rural) on pain management, outcomes, or experience and (3) were original research articles, qualitative studies, quantitative and observational studies (cross‐sectional/longitudinal). Definitions of rurality vary globally and are often country‐specific due to the geographical and population spread. In this study, we included all studies that described differences in urban/rural areas and described each individual study setting.

We excluded studies if they (1) focused on children or adolescents, (2) did not investigate the influence of place of residence (urban or rural) on pain management, outcomes or experiences, (3) were systematic reviews, editorials, letters, conference proceedings, and intervention studies, and (4) were published in a language other than English. Intervention studies were excluded because the review sought to characterise differences in pain management under routine care. Interventions may alter care delivery and outcomes, preventing attribution of observed differences to the rural or urban healthcare context itself. All publication dates were included. No unpublished studies or grey literature were included.

### 2.3 Screening

Duplicates were removed. Dual title/abstract screening was conducted independently against the inclusion criteria by four authors. This was followed by independent full‐text dual assessment of the short‐listed full texts against the eligibility criteria by two authors. Any disagreements were resolved by a third author.

### 2.4 Study Quality Assessment

All included articles were appraised to ensure the reliability and replicability of their findings. Observational studies were appraised using the Newcastle Ottawa Scale (NOS) (Wells et al., n.d.), qualitative studies using the Critical Appraisal Skills Programme (CASP) checklist (CASP 2025), and any mixed‐method studies were appraised using the Mixed Methods Appraisal Tool (MMAT) (Hong et al. 2018). Each study was assessed for risk of bias independently by two reviewers and given a rating of poor, fair, or good. Any disparities in the ratings were discussed with a third author, and consensus was reached. No studies were excluded based on quality assessment to allow all relevant findings to be explored in the narrative synthesis.

### 2.5 Data Extraction and Synthesis

Key data extracted from each paper included: first author, publication year, country of publication, sample size, study design, objectives and definitions of rurality. The pain management interventions and/or services, pain outcomes, patient experiences of pain, and any specific access issues related to rurality were also recorded.

A narrative approach was taken to synthesise the findings in the included studies, because the studies were heterogeneous in terms of populations, outcomes and design and were not suitable for meta‐analysis. A narrative synthesis was a flexible and inclusive approach that allowed the wide range of included studies to be synthesised.

## 3 Results

Database searching identified 2686 records, shown in a PRISMA flowchart in Figure 1 of which 345 were duplicates and were removed. Five records were identified from the authors' personal library and reference list searching. After title/abstract screening, 2265 records were excluded and a total of 74 records were retrieved for full‐text screening.

**FIGURE 1:**
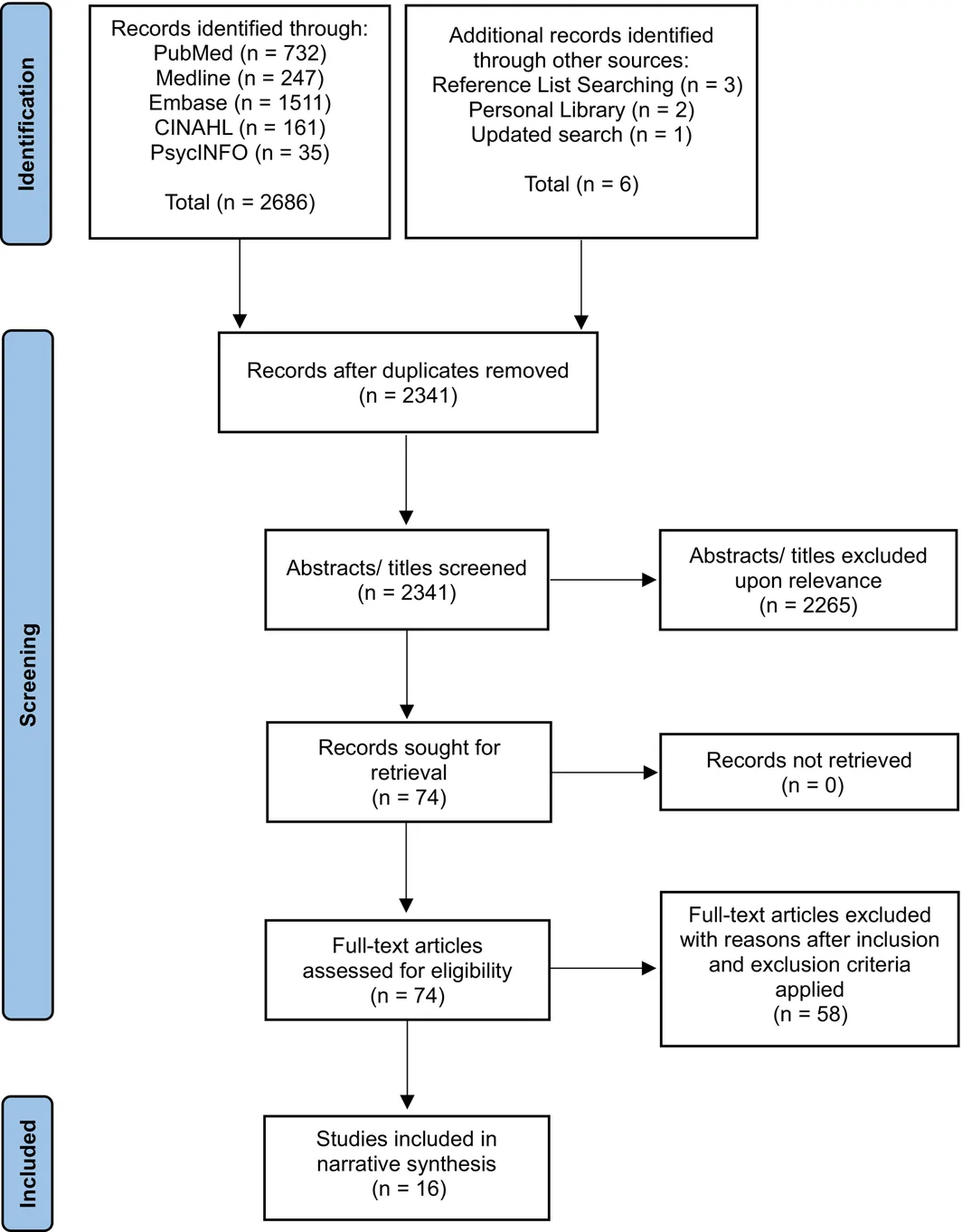
PRISMA flowchart detailing study identification and selection process.

### 3.1 Characteristics of Included Studies

The main characteristics of the 16 included studies are summarised in Table 1. All studies were published between 1996 and 2025. One study was conducted in Spain, two in Australia, six in the UK and seven in the USA.

Twelve observational studies (Bath et al. 2018; Brockopp et al. 1996; Carey et al. 1996; Catalá et al. 2021; Glenister et al. 2024; Goode et al. 2013; Hadlandsmyth et al. 2023; Hoffman et al. 2002; Mares et al. 2023; Prunuske et al. 2014; Rafferty et al. 2021; Tripp et al. 2006), three qualitative studies (Briggs et al. 2012; Grant et al. 2025; Jebara et al. 2023), and one mixed‐methods study (Currie et al. 2015) were included. The sample sizes ranged from 14 participants (Briggs et al. 2012; Jebara et al. 2023) to 9,325,603 participants (a study using routinely collected health data) (Prunuske et al. 2014). Qualitative studies had smaller sample sizes compared to quantitative studies (as illustrated in Table 2).

**TABLE 2:** Characteristics of included studies.

First author (year)	Country of publication	Sample size	Type of study	Definitions of rurality	Objectives/aim of study
Bath et al. (2018)	UK	113,647	Cross‐sectional	“Rural” communities; sub‐groups or MIZ based on the size of commuting flows to any larger urban centre	To explore how adults with chronic back disorders use primary care, chiropractic, and physiotherapy services To describe sociodemographic and other factors linked to different healthcare providers in adult Canadians with chronic back disorders
Briggs et al. (2012)	UK	14	Qualitative	Rural residents: people living in Kununurra, Albany and Kalgoorlie	To investigate barriers faced by rural individuals in Western Australia in accessing information and services, as well as adopting implementing effective self‐management practises for CLBP
Brockopp et al. (1996)	UK	125	Cross‐sectional	Rural town; small town largely dependent on coal mining; data collected in the mountainous part of the state	To assess the attitudes, knowledge and management of acute pain of an elderly population to identify potential barriers that impact pain management
Carey et al. (1996)	USA	8067	Cross‐sectional	“Counties organised by percentage of the population residing in urban areas; those below the median were considered rural”	To evaluate the prevalence of low back pain and what factors influence care‐seeking in a specific population
Catalá et al. (2021)	Spain	234	Cross‐sectional	Defined by the Publication of Urban Areas in Spain 2019, by the Ministry of Transport, Mobility and Urban Agenda, DG of Housing and Floor	To investigate whether place of residence (urban or rural) impacts the symptoms related to fibromyalgia To investigate whether place of residences affects disease acceptance and its impact on emotional, physical and cognitive symptoms
Currie et al. (2015)	UK	168	Mixed‐methods	Scottish Government's Urban–rural classification: number of residents is < 3000	To investigate behaviours towards, current utilisation of and willingness to accept utilisation of technology in healthcare
Glenister et al. (2024)	Australia	2680	Cross‐sectional	Australian Statistical Geography Standard—Remoteness Areas MMM classifications: MM3: large rural town MM4: medium rural town	To determine chronic pain prevalence and specialist pain clinics utilisation in rural areas To explore factors related to adequately controlled pain
Grant et al. (2025)	Australia	15 HCPs 27 Consumers	Qualitative	Australian Statistical Geography Standard—Remoteness Areas MMM classifications	To assess what HCPs are rural consumers consulting for chronic pain care To investigate how rural consumers access chronic pain services To determine what referral pathways rural consumers, use for chronic pain care To assess how HCPs manage chronic pain in rural Australia To compare consumer and HCP reports of how pain is managed in rural Australia to guideline recommendations for chronic pain care
Goode et al. (2013)	USA	558	Cross‐sectional	U.S. Department of Agriculture's Rural–Urban Continuum Codes (RUCC). codes 1–3: urban codes 4–9: rural	To describe the demographic and health‐related characteristics among rural/urban individuals with CLBP To ascertain whether there are variations in the usage of diagnostics and treatments for CLBP between rural and urban individuals To ascertain whether there is a correlation between rurality and healthcare provision and whether correlations vary based on race or gender
Hadlandsmyth et al. (2023)	USA	2,261,030	Cohort	Rural status defined using RUCA; “mapped by the census tract or zip code of the veteran's residence. Urban RUCA codes: 1.0, 1.1, 2.0, 2.1, 3.0, 4.1, 5.1, 7.1, 8.1, and 10.1 (others were rural)”	To assess variations in pain management between rural and urban women Veterans To ascertain if the impact of rurality varied between women and men
Hoffman et al. (2002)	USA	188	Cross‐sectional	U.S. Census Bureau (1995) definition of a rural area: number of residents is < 2500	To determine the prevalence and reported experience of chronic pain in North Dakota To compare the prevalence and characteristics of chronic pain across rural and urban individuals To assess the effect of chronic pain on quality of life To understand the impact of social support on chronic pain
Jebara et al. (2023)	UK	14	Qualitative	UK Rural–Urban Classification: number of residents is < 3000 people, and with a drive time of over 30 min to a settlement of ≥ 10,000	To investigate and understand the perspectives, experiences, and factors influencing the behaviour of older individuals concerning the management of chronic pain in remote and rural areas in the Scottish Highlands
Mares et al. (2023)	USA	2,135,216	Cross‐sectional	Rural status defined using RUCA; “mapped by the census tract or zip code of the veteran's residence. Urban RUCA codes: 1.0, 1.1, 2.0, 2.1, 3.0, 4.1, 5.1, 7.1, 8.1, and 10.1 (others were rural)”	To isolate variations in Veterans Affairs chronic pain care among Black, Asian, and Hispanic Americans, compared to non‐Hispanic White Americans, and assess how rurality and race overlap
Prunuske et al. (2014)	UK	9,325,603	Cross‐sectional	Rural residents: “people living either within an MSA that had no centre city or outside an MSA”	To assess disparities in rural and non‐rural differences opioid prescribing numbers for managing CNMP
Rafferty et al. (2021)	USA	3598	Cross‐sectional	North Carolina Rural Centre's county‐based classification—rural countries: 78 countries with average population density of ≤ 250 individuals	To describe chronic pain prevalence, severity, causes, and management strategies utilised by North Carolina individuals in rural, suburban, and urban localities
Tripp et al. (2006)	USA	1069	Cross‐sectional	Statistics Canada classifications for census Subdivision: Townships: rural Towns: town Cities: urban	To determine the prevalence of chronic pain in the south‐eastern region of Ontario Assess whether the reporting of pain can be anticipated by factors outlined in prior studies (i.e., age, residence, income and health status)

Abbreviations: CLBP, chronic low back pain; HCP, healthcare professional; MIZ, metropolitan influenced zones; MMM, Modified Monash Model; MSA, metropolitan statistical area; RUCA, Rural Urban Commuting Areas.

Twelve studies described pain management (Bath et al. 2018; Carey et al. 1996; Currie et al. 2015; Goode et al. 2013; Hadlandsmyth et al. 2023; Hoffman et al. 2002; Jebara et al. 2023; Mares et al. 2023; Prunuske et al. 2014; Rafferty et al. 2021; Tripp et al. 2006), eight studies investigated pain‐related outcomes (Briggs et al. 2012; Catalá et al. 2021; Goode et al. 2013; Hadlandsmyth et al. 2023; Hoffman et al. 2002; Jebara et al. 2023; Rafferty et al. 2021; Tripp et al. 2006), and five studies explored patient experiences (Briggs et al. 2012; Brockopp et al. 1996; Hoffman et al. 2002; Jebara et al. 2023). The studies assessed pain management techniques, including the use of opioids and other medications, as well as comparisons between access to primary care and specialist healthcare services between rural and urban adults. The main outcomes considered were pain intensity and quality of life. With respect to potential examples of rural population disadvantage, nine studies (Bath et al. 2018; Briggs et al. 2012; Glenister et al. 2024; Goode et al. 2013; Hadlandsmyth et al. 2023; Jebara et al. 2023; Mares et al. 2023; Rafferty et al. 2021) investigated specific access barriers related to rurality, whereas ten studies explored other examples of rural population disparities (Bath et al. 2018; Briggs et al. 2012; Currie et al. 2015; Glenister et al. 2024; Goode et al. 2013; Hadlandsmyth et al. 2023; Jebara et al. 2023; Mares et al. 2023; Rafferty et al. 2021), for example prohibitive costs (Glenister et al. 2024).

### 3.2 Definitions of Geographical Location

Fifteen out of the 16 studies (Bath et al. 2018; Briggs et al. 2012; Brockopp et al. 1996; Carey et al. 1996; Catalá et al. 2021; Currie et al. 2015; Glenister et al. 2024; Grant et al. 2025; Hadlandsmyth et al. 2023; Hoffman et al. 2002; Jebara et al. 2023; Mares et al. 2023; Prunuske et al. 2014; Rafferty et al. 2021; Tripp et al. 2006) included a definition of rurality. Hadlandsmyth et al. (2023) and Mares et al. (2023) used the same definitions of rurality according to the Rural–Urban Commuting Areas (RUCA) (USDA 2023) and study participants were in both cases military veterans. Prunuske et al. (2014) and Bath et al. (2018) classified participants according to metropolitan and micropolitan statistical areas, using these classifications to distinguish geographical location. Six studies (Catalá et al. 2021; Currie et al. 2015; Glenister et al. 2024; Hoffman et al. 2002; Jebara et al. 2023; Rafferty et al. 2021) used published definitions of rurality, which were defined by the respective countries of publication (see Table 2).

### 3.3 Results of Included Studies

The findings of each study are summarised in Table 3.

**TABLE 3:** Summary of findings of included studies.

First author (year)	Subgroup analysis of pain management	List of healthcare services accessed and pharmacological and/or non‐pharmacological management approaches	Pain outcomes	Patient experiences	Examples of rural population disadvantage (if any)	Specific access issues related to rurality
Bath et al. (2018)	No statistical difference in use of primary care or chiropractor services Urban residents more likely to use physiotherapists	Primary care Chiropractor Physiotherapist			Rural residents 30% more likely to experience chronic back disorders	Challenges in accessing healthcare services
Briggs et al. (2012)		Self‐management Primary care	Psychological burden	Feelings of isolation and frustration of rural residents Inadequate knowledge and skills among GPs Competing lifestyle demands	Shortage of health resources	Challenges in accessing: Information Healthcare services long distances
Brockopp et al. (1996)				Similar attitudes of rural and urban adults Rural residents show more apprehension than urban about taking pain medications and caring of themselves if they experience pain, and are hospitalised Rural people felt that: “they could handle pain better” as they got older compared to urban		
Carey et al. (1996)	No association between care‐seeking and place of residence (urban or rural)	Chiropractic care Doctor care				
Catalá et al. (2021)			Rurality associated with: Higher pain scores Lower physical and mental fatigue scores Similar outcomes in anxiety, depression, and functional limitations		Shortage of health resources	
Currie et al. (2015)	Rural residents less likely to use technology as part of their healthcare	Using technology			Slow, unreliable broadband services	
Glenister et al. (2024)		Treatments: Prescription narcotics Over‐the‐counter medications Heath care services: Primary care Specialty care Allied health Complementary and alternative medicine				Challenges in accessing healthcare services: Long distances Long waiting times Prohibitive costs
Grant et al. (2025)	Rural residents: Less likely to be able to access pain care services More likely to be prescribed opioids and other medications including cannabinoids	Treatments: Opioids Analgesics Heath care services: Primary care Specialty care Allied health Complementary and alternative medicine Multidisciplinary pain services Emergency departments Telehealth		Consumers reported receiving care that did not align with guidelines Negative experiences with HCP Limited access to telehealth	Poor connectivity, technology challenges and digital literacy Consumers doing their own research to find services that support their pain management	Challenges in accessing pain care services
Goode et al. (2013)	Rural residents prescribed more opioids No statistically significant differences in diagnostic imaging, use of providers, medications, and physical treatments among rural and urban residents	Physical treatments: exercise and massage Medications: NSAIDS and narcotics Care by providers i.e., rheumatologists	Rurality associated with: Poorer health including depression Being uninsured Lower use of health care services Poorer physical function Higher functional limitations		Health insurance status: rural residents being uninsured were unable to afford costs of specialised care	Challenges in accessing healthcare services: Prohibitive costs
Hadlandsmyth et al. (2023)	Rural women: Prescribed more opioids and nonopioids More likely to receive primary care and less likely to receive specialty care (e.g., emergency care)	Opioids Nonopioids (NSAIDS, muscle relaxants, gabapentin, duloxetine and benzodiazepines) Primary care Specialty care (physical therapy, pain clinic, mental health, acupuncture, chiropractic, massage) ED/urgent care	Rural veterans: Higher levels of comorbidities		Disparities in access to specialist pain clinic with higher numbers of prescribed opioid in rural areas	Challenges in accessing: Healthcare services Nonmedication therapies
Hoffman et al. (2002)	More rural residents using oral medications and undergoing surgery Less rural residents using specialty clinics including visiting chiropractors, physical therapists and massage therapists	Oral medication Chiropractic care Physical therapy Surgery Massage therapy	Quality of life scores among urban and rural residents were similar Rural groups experiencing pain of greater intensity	More urban residents reported: They are “strong enough to handle their pain without a doctor” They “can't pay for medical treatment” More rural residents said: “can't take time off work”		
Jebara et al. (2023)	No significant difference in the usage of pharmacological and non‐pharmacological interventions between rural and urban residents.	Pharmacological approach: opioids and nonopioids Nonpharmacological approach: exercise, physio, chiropractor, acupuncture, heat pad	No difference in pain intensity due to rurality	Negative impact on daily lives of all residents; no rural difference Shared need for enhancement of pain management		Challenges in accessing healthcare services: Long waiting times Long distances
Mares et al. (2023)	No differences in primary care visits between rural and urban residents. More urban residents visited physical therapist compared to rural residents less rural residents visited pain clinics and received mental health care for specific racial rural groups compared to their urban counterparts	Primary care Pain clinic Physical therapy Complementary care Mental health clinic ED/urgent care			Provider shortages	Challenges in accessing healthcare services: Long distances Travel time Long waiting times
Prunuske et al. (2014)	Rural residents more likely to be prescribed opioids	Opioids Other medications				
Rafferty et al. (2021)	Rural residents: Used less non‐medication treatments Were less likely to use multiple treatments for chronic pain	Over‐the‐counter medication Prescription narcotics Prescription non‐narcotics Nonpharmacological approach Alcohol, marijuana street drugs	Pain frequency and severity not associated with rurality		Shortage of health resources and medical specialists	Challenges in accessing nonmedication therapies
Tripp et al. (2006)	Rural residents: Less likely to utilise healthcare services More likely to use medications	Healthcare services Medications	Rurality associated with: Increasing severity of pain Lower health status Depression			

Abbreviations: ED, emergency department; GP, general practitioner; HCP, healthcare professional; NSAID, nonsteroidal Anti‐inflammatory Drug.

#### 3.3.1 The Impact of Place of Residence (Urban or Rural) on Management of CNMP

Twelve studies (Bath et al. 2018; Carey et al. 1996; Currie et al. 2015; Goode et al. 2013; Grant et al. 2025; Hadlandsmyth et al. 2023; Hoffman et al. 2002; Jebara et al. 2023; Mares et al. 2023; Prunuske et al. 2014; Rafferty et al. 2021; Tripp et al. 2006) examined pain management strategies among individuals with CNMP in relation to place of residence. There was a general agreement in all studies that rural residents were more likely to access primary care services for pain management than specialist services compared to their urban counterparts. While Bath et al. (2018) and Mares et al. (2023) reported no significant differences in primary care visitation rates for CNMP between rural and urban populations; both studies found that urban residents had a higher frequency of specialist clinic visits.

Tripp et al. (2006) noted that rural residents utilised healthcare services for pain management less frequently overall, which the authors attributed to limited availability of non‐pharmacological treatment options in rural areas. Rafferty et al. (2021) reported that urban adults were significantly more likely to receive physical therapy for pain management (urban 42.6%, suburban 22.3%, rural 19.8%, *p* < 0.001). Similar patterns were observed in studies by Hoffman et al. (2002) and Rafferty et al. (2021).

Five studies (Goode et al. 2013; Grant et al. 2025; Hadlandsmyth et al. 2023; Prunuske et al. 2014; Rafferty et al. 2021) investigated the use of prescription opioids for CNMP management. These studies consistently reported that rural residents were more likely to be prescribed opioids compared to their urban counterparts. Four out of six studies (Hadlandsmyth et al. 2023; Hoffman et al. 2002; Rafferty et al. 2021; Tripp et al. 2006) reported a statistically significant association between rural residence and greater reliance on pharmacological treatments for CNMP, including opioid and non‐opioid medications. However, two studies (Goode et al. 2013; Jebara et al. 2023) conducted in USA and the UK respectively, found no statistically significant differences in medication usage between urban and rural populations. The study by Goode et al. (2013) found slightly higher prescription rates rural settings but the differences in prescribing between urban/rural areas did not reach statistical significance.

Grant et al. (2025) highlighted a disparity between healthcare professionals' perceptions and patients' experiences in rural settings. While clinicians reported delivering care aligned with clinical guidelines, rural patients frequently described treatment practices that deviated from these guidelines, including overprescription of opioids and cannabinoids. The authors further identified a disconnect between care received by rural patients and existing clinical guidelines for CNMP management.

Cost and insurance coverage were identified as barriers to equitable pain management. Goode et al. (2013) (study conducted in USA) and Glenister et al. (2024) (study conducted in Australia), noted that non‐pharmacological therapies were not consistently covered by health insurance or national healthcare systems, in country and country, respectively. As a result, patients in rural areas often incurred out‐of‐pocket expenses, such as travel costs and lost wages which may have restricted their access to such treatments.

#### 3.3.2 The Impact of Place of Residence (Urban or Rural) on Outcomes of People With CNMP

Eight studies (Briggs et al. 2012; Catalá et al. 2021; Goode et al. 2013; Hadlandsmyth et al. 2023; Hoffman et al. 2002; Jebara et al. 2023; Rafferty et al. 2021; Tripp et al. 2006) examined outcomes among individuals with CNMP in relation to place of residence. The evidence was predominantly derived from cross‐sectional studies, with outcomes generally assessed at a single point. Only one included study (Hadlandsmyth et al. 2023) used a cohort design to examine outcomes over time. Consequently, the findings primarily reflect differences between rural and urban populations at the point of assessment and provide limited evidence regarding changes in outcomes over time.

Findings regarding outcomes were heterogeneous. Several studies reported poorer outcomes among rural residents with CNMP, including greater symptom burden, higher pain intensity, poorer psychological wellbeing, and greater challenges accessing appropriate support and self‐management resources (Briggs et al. 2012; Catalá et al. 2021; Goode et al. 2013; Hadlandsmyth et al. 2023; Hoffman et al. 2002; Tripp et al. 2006), However, differences were not consistently observed across all outcomes. Brockopp et al. (1996) reported comparable pain management knowledge and attitudes between populations, while Hoffman et al. (2002) identified rural–urban differences in pain prevalence and intensity but no significant differences for some secondary outcomes, including aspects of quality of life and social support. Qualitative studies further highlighted the impact of rurality on experiences of care, including barriers related to access, information and self‐management support.

Psychological outcomes also differed by place of residence. Three studies (Catalá et al. 2021; Goode et al. 2013; Tripp et al. 2006) reported poorer mental wellbeing among rural residents, with two studies (Goode et al. 2013; Tripp et al. 2006) identifying significantly higher rates of depression in rural residents with CNMP compared to urban residents with CNMP.

#### 3.3.3 The Impact of Place of Residence (Urban or Rural) on Experiences of People With CNMP

Nine studies (Bath et al. 2018; Briggs et al. 2012; Glenister et al. 2024; Goode et al. 2013; Grant et al. 2025; Hadlandsmyth et al. 2023; Jebara et al. 2023; Mares et al. 2023; Rafferty et al. 2021) identified that rural residence posed a significant barrier to accessing healthcare services for CNMP. Jebara et al. (2023) emphasised that participants from both rural and urban areas experienced challenges with current pain management approaches; however, rural participants described additional barriers, including limited access to specialists, long travel distances, and fewer local resources. Briggs et al. (2012) described how rural individuals felt overlooked by healthcare professionals, often relying on self‐management strategies and informal support networks to cope with persistent pain. Similarly, Grant et al. (2025) reported that rural participants experienced frustration and isolation due to limited access to information and healthcare services, compounding the psychosocial burden of chronic pain.

Despite healthcare professionals reporting guideline‐informed practices, many patients perceived their care as misaligned with best practice. Similarly, Currie et al. (2015) reported that many rural participants described their therapeutic relationships with healthcare professionals as negative and suggested a need for improved communication, continuity, and trust in provider–patient interactions in rural settings. Hoffman et al. (2002), noted that rural residents in USA more frequently reported difficulty in taking time off work to attend appointments or engage in recommended pain management strategies (e.g., attending pain clinics or physical therapy appointments) compared to those living in urban areas.

### 3.4 Risk of Bias and Quality Assessment of Included Studies

Ten studies were rated as fair quality and six as good quality as shown in Table S1. Common methodological issues that were noted in the studies included: unsatisfactory response rate (Bath et al. 2018; Catalá et al. 2021; Glenister et al. 2024; Goode et al. 2013; Rafferty et al. 2021) and use of a non‐validated screening tool for outcomes (Bath et al. 2018; Goode et al. 2013; Prunuske et al. 2014; Rafferty et al. 2021).

## 4 Discussion

### 4.1 Main Findings

This review highlights inequalities in the management of CNMP between rural and urban areas. Rural residence was generally associated with reduced access to specialist pain management services and non‐pharmacological approaches. Several studies highlighted higher use of pharmacological treatments, including opioid and non‐opioid medications (Goode et al. 2013; Grant et al. 2025; Hadlandsmyth et al. 2023; Hoffman et al. 2002; Prunuske et al. 2014; Rafferty et al. 2021; Tripp et al. 2006) among rural residents.

Findings regarding outcomes were less consistent, but the overall evidence suggests that geographic location may influence the experience and management of CNMP. Differences in pain intensity, symptom burden, psychological wellbeing, and self‐management may reflect cumulative disparities in healthcare access, availability of supportive services, and opportunities for ongoing pain management. However, these associations were not consistent across all outcomes, highlighting the complexity of the relationship between rurality and pain‐related outcomes. Differences between studies may reflect variation in healthcare systems, population characteristics, definitions of rurality, outcome measures, and availability of pain management resources.

Several interacting mechanisms may explain how geographic location could influence CNMP management. These include long travel distances, waiting times, and occupational demands that can make attending appointments difficult for rural patients. Qualitative evidence (Grant et al. 2025) further highlighted feelings of frustration, isolation, inadequate support, and lower satisfaction with clinical interactions among rural people with CNMP.

Importantly, urban residents also experience significant challenges in accessing adequate pain management (Brockopp et al. 1996; Hoffman et al. 2002; Jebara et al. 2023) suggesting that improving access to holistic pain management is required across the wider population. No studies identified clear benefits of rural residence in relation to CNMP management, outcomes, or experiences. Nonetheless, these findings should be interpreted in the context of variation in methodological quality, study design, definitions of rurality and outcome measurement.

### 4.2 Context Within Wider Literature

Our findings align with existing literature associating rurality with poorer health outcomes among individuals with CNMP, including higher pain intensity and reduced physical functioning (Catalá et al. 2021; Hoffman et al. 2002; Jebara et al. 2023; Tripp et al. 2006). Limited access to multidisciplinary and non‐pharmacological approaches may contribute to greater reliance on pharmacological treatments, including both opioid and non‐opioid medications (Hadlandsmyth et al. 2023; Hoffman et al. 2002; Rafferty et al. 2021; Tripp et al. 2006). This may reflect healthcare system constraints rather than patient preference, with rural areas often having fewer specialist services, rehabilitation programmes, and psychological therapies. Longer travel distances and reduced availability of specialist pain services may further restrict opportunities to engage with multidisciplinary models of care (Bath et al. 2018; Briggs et al. 2012; Glenister et al. 2024; Goode et al. 2013; Grant et al. 2025; Hadlandsmyth et al. 2023; Jebara et al. 2023; Mares et al. 2023; Rafferty et al. 2021).

In rural areas with limited healthcare infrastructure, primary care providers may assume responsibilities typically shared with specialists.

Rural residents experiencing pain rely more heavily on primary care and have reduced access to specialist services, a pattern also observed in other chronic conditions such as hypertension, cardiovascular disease, and asthma (Bath et al. 2018; Briggs et al. 2012; Glenister et al. 2024; Goode et al. 2013; Grant et al. 2025; Hadlandsmyth et al. 2023; Jebara et al. 2023; Mares et al. 2023; Rafferty et al. 2021).

Medication‐centred approaches may diverge from international guidance recommending a biopsychosocial and multidisciplinary approach to pain management (NICE 2021; Smalley et al. 2010). The risks associated with opioid use are particularly relevant given evidence from the USA, where opioid‐related mortality rates in rural regions have surpassed urban areas since the mid‐2000s, with particularly high mortality rates reported in rural Appalachia, North Carolina, and California (Keyes et al. 2014; Monnat and Rigg 2016). Geographic isolation, travel distances, and waiting times may therefore contribute to medication‐based self‐management as a practical response to limited access to alternative forms of care (Bath et al. 2018; Briggs et al. 2012; Glenister et al. 2024; Goode et al. 2013; Hadlandsmyth et al. 2023; Jebara et al. 2023; Mares et al. 2023; Rafferty et al. 2021).

The geographic inequalities observed in CNMP management reflect broader patterns of rural healthcare disadvantage. Rural populations with diabetes may experience delayed diagnosis and reduced access to specialist care and self‐management education (Trivedi et al. 2011). Similarly, rural mental health services may be limited by reduced availability, stigma, and logistical barriers, resulting in reliance on pharmacological treatments rather than psychological therapies (Roberts et al. 2018; Smalley et al. 2010). Comparable disparities have been reported in asthma, where reduced access to respiratory specialists and pulmonary rehabilitation may contribute to poorer outcomes (AIHW 2010; Purdy et al. 2011).

Limited availability and affordability of non‐pharmacological treatments, including acupuncture, massage, and yoga, were also identified. In a US‐based study, rural residents reported that such interventions are often not covered by insurance and may involve substantial out‐of‐pocket costs (Goode et al. 2013). Economic disadvantage may therefore compound geographic inequalities. Rural patients can face direct costs such as travel expenses and indirect costs through lost income from attending appointments or being unable to work because of chronic pain (Pong et al. 2011). Rural populations may also have substantially higher healthcare expenditure than urban populations (Khan et al. 2017; Li et al. 2014). However, the full economic burden of rurality and its relationship with patient outcomes remain poorly understood.

Digital health approaches may provide an opportunity to address some geographic barriers. The selfBACK randomised controlled trial (Sandal et al. 2021) demonstrated that an AI‐driven smartphone decision‐support system significantly improved pain‐related disability in individuals with chronic low back pain compared to standard care. Such technologies could decentralise care, reduce travel and provide remote access to pain specialists and self‐management interventions. However, their effectiveness in rural populations depends on digital accessibility, which may vary geographically.

### 4.3 Implications for Policy and Practice

Difficulty accessing centralised, specialist services is a recurring theme in the rural CNMP literature. Digital self‐management tools could extend the reach of chronic pain services, but unreliable broadband and limited digital access may restrict their uptake in rural communities (Currie et al. 2015). Improving digital infrastructure should therefore form part of wider strategies to reduce geographic inequalities.

Improving community and primary care capacity may also improve access to evidence‐based CNMP management where specialist services are geographically concentrated. Rural residents expressed concerns regarding GPs' pain‐specific knowledge and the limited availability of local pain management resources (Briggs et al. 2012; Grant et al. 2025). Primary care providers should be supported through enhanced training, resources, and clinical guidance, particularly where they may be the main provider of pain care.

Affordability is another barrier to accessing non‐pharmacological treatment (Goode et al. 2013). Given the high prevalence of comorbidities among people with CNMP (Keyes et al. 2014), integrating pain management with existing care pathways for commonly concurring chronic conditions may improve continuity and efficiency of healthcare.

### 4.4 Strengths and Limitations

This review spanned several databases and identified a wide range of quantitative, qualitative, and mixed‐methods research, based on a pre‐specified protocol (Stylianou et al. 2024). Multiple authors were involved in study screening and risk‐of‐bias assessment, strengthening the review process. Inclusion of both quantitative and qualitative evidence enabled consideration of measurable differences in management and healthcare utilisation alongside patient experiences and perceived barriers.

However, most included studies were of fair methodological quality, with limitations including unsatisfactory response rates and use of non‐validated outcome measures.

Substantial heterogeneity in populations, definitions of rurality, outcome measures and healthcare systems prevented meta‐analysis and limited the ability to generate pooled estimates of the magnitude of geographic inequalities.

The research was restricted to English, potentially excluding relevant studies published in other languages. Included studies were conducted in the USA, Australia and Europe, with no representation from low‐ or middle‐income settings, limiting generalisability. Variation in socioeconomic circumstances, education, ethnicity and other markers of disadvantage also limits conclusions about whether findings apply equally across population groups. Additionally, rural populations are heterogeneous, and findings may not be transferable between settings with different healthcare infrastructure, geography and socioeconomic contexts.

Finally, definitions of rurality varied between studies, often using national census criteria based on population size or density. These inconsistencies make direct comparisons between countries difficult and may contribute to differences in reported findings.

## 5 Conclusions

Rural residents are more likely to be managed in primary care and with opioid‐based treatments for CNMP management than urban residents, potentially reflecting geographic barriers including long travel distances, limited specialist pain services and reduced access to non‐pharmacological interventions. Evidence regarding outcomes was less consistent; however, several studies suggested that rural residents may experience greater pain severity, reduced quality of life and a higher burden of comorbidities. Qualitative evidence also indicated more negative experiences of pain care, including reduced perceived support, limited information and challenges accessing appropriate services. These findings should be interpreted in light of methodological limitations and substantial heterogeneity between studies, particularly in definitions of rurality and outcome measures. Nevertheless, the evidence highlights the need for targeted policy initiatives and service reconfiguration to reduce geographic inequalities and ensure equitable, evidence‐based CNMP management regardless of place of residence.

## Author Contributions

**Christiana Stylianou:** formal analysis, investigation, methodology, project administration, writing – original draft, writing – review and editing. **Rosalind Adam:** conceptualization, formal analysis, supervision, writing – review and editing. **Fortune Kwushue:** formal analysis, writing – review and editing. **Barbara I. Nicholl:** conceptualization, formal analysis, supervision, writing – review and editing.

## Funding

R.A. is funded by a Senior Clinical Academic Fellowship from the Chief Scientist Office (CSO), Scottish Government Health and Social Care Directorate (SCAF/18/02). The funder did not have any input into the design of this study or the analysis.

## Conflicts of Interest

The authors declare no conflicts of interest.

## Supporting information


**Appendix S1:** Search Strategy used for database Medline for systematic review.
**Table S1:** Quality appraisal of included studies NOS (Newcastle‐Ottawa Scale) is scored out of 9, and the CASP qualitative checklist is scored out of 10; higher scores indicate higher methodological quality.
