# Supplementary material for: How Does Place of Residence Influence the Experience and Management of Chronic Non‐Malignant Pain? A Systematic Review
Source: Eur J Pain. 2026 Nov 2;30(10):e70443. doi: 10.1002/ejp.70443 (PMC13726444; doi:10.1002/ejp.70443)
Supplement: Supplementary file 1 [file EJP-30-0-s001.docx]

**AppendixS1: Search Strategy used for database Medline for systematic review.**

Search terms for Medline (via Ovid): 1946 to 2^nd^ April 2025

1. Analgesics, Opioid/ or Pain Management/
2. ((pain adj2 manag*) or (opiod* adj2 analgesi*)).ti,ab.
3. 1 or 2
4. Chronic Pain/
5. (chronic* adj2 pain*).ti,ab.
6. 4 or 5
7. 3 or 6
8. Rural Health/
9. exp Rural Health Services/
10. (rural adj2 health).ti,ab.
11. 8 or 9 or 10
12. Urban Health/
13. Urban Health Services/
14. (urban adj2 health).ti,ab.
15. 12 or 13 or 14
16. 7 and 11
17. 7 and 15
18. 16 or 17

*(247 records found)*

**TableS1:**

Quality Appraisal of Included Studies

NOS (Newcastle-Ottawa Scale) is scored out of 9, and the CASP qualitative checklist is scored out of 10; higher scores indicate higher methodological quality.

| **First author**  **(year)** | **Quality Assessment tool** | **Quality Assessment Score** | **Quality of Study** |
| --- | --- | --- | --- |
| Bath et al.  (2018) (Bath et al., 2018) | NOS | 6 | fair |
| Briggs et al.  (2012) (Briggs et al., 2012) | CASP qualitative checklist | 10 | good |
| Brockopp et al.  (1996) (Brockopp D et al., 1996) | NOS | 5 | fair |
| Carey et al.  (1996) (Carey TS et al., 1996) | NOS | 7 | good |
| Catala et al.  (2021) (Catalá P et al., 2021) | NOS | 6 | fair |
| Currie et al.  (2015) (Currie M et al., 2015) | MMAT checklist | n/a  addressed all quality criteria | good |
| Glenister et al.  (2024) (Glenister K et al., 2024) | NOS | 7 | good |
| Grant et al. (2025) (Grant et al., 2025) | CASP qualitative checklist | 10 | good |
| Goode et al.  (2013) (Goode AP et al., 2013) | NOS | 7 | good |
| Hadlandsmyth et al. (2023) (Hadlandsmyth et al., 2023) | NOS | 8 | good |
| Hoffman et al.  (2002) (Hoffman PK et al., 2002) | NOS | 6 | fair |
| Jebara et al.  (2023) (Jebara T et al., 2023) | CASP qualitative checklist | 10 | good |
| Mares et al.  (2023) (Mares JG et al., 2023) | NOS | 7 | good |
| Prunuske et al.  (2014) (Prunuske JP et al., 2014) | NOS | 7 | good |
| Rafferty et al.  (2021) (Rafferty AP et al., 2021) | NOS | 6 | fair |
| Tripp et al.  (2006) (Tripp DA et al., 2006) | NOS | 5 | fair |

Abbreviations: NOS, Newcastle Ottowa Scale; CASP, Critical Appraisal Skills Programme; MMAT, Mixed Methods Appraisal Tool; n/a, not applicable
